# Chronic Hepatitis C Virus Infection, Extrahepatic Disease and the Impact of New Direct-Acting Antivirals

**DOI:** 10.3390/pathogens13040339

**Published:** 2024-04-19

**Authors:** Nahum Méndez-Sánchez, Carlos E. Coronel-Castillo, Mariana Michelle Ramírez-Mejía

**Affiliations:** 1 Unit Liver Research, Medica Sur Clinic & Foundation, Mexico City 14050, Mexico; mich.rm27@gmail.com; 2 Faculty of Medicine, National Autonomous University of Mexico, Mexico City 04510, Mexico; 3 Internal Medicine Section, Central Military Hospital, Mexico City 11649, Mexico; carlos-cc@outlook.com; 4 Plan of Combined Studies in Medicine (PECEM MD/PhD), Faculty of Medicine, National Autonomous University of Mexico, Mexico City 04510, Mexico

**Keywords:** Hepatitis C virus, direct-acting antivirals, mixed cryoglobulinemia, non-Hodgkin lymphoma, insulin resistance, lipids

## Abstract

Chronic hepatitis C virus infection is an important cause of liver cirrhosis, hepatocellular carcinoma and death. Furthermore, it is estimated that about 40–70% of patients develop non-hepatic alterations in the course of chronic infection. Such manifestations can be immune-related conditions, lymphoproliferative disorders and metabolic alterations with serious adverse events in the short and long term. The introduction of new Direct-Acting Antivirals has shown promising results, with current evidence indicating an improvement and remission of these conditions after a sustained virological response.

## 1. Introduction

Despite antiviral therapy, viral hepatitis infections are an important cause of liver cirrhosis and hepatocellular carcinoma (HCC). According to the World Health Organization (WHO), by 2021, up to 63% of individuals with liver cirrhosis had experienced infection either by the hepatitis B virus (HBV) or hepatitis C virus (HCV). Of that number, HCV infection accounts for 21% of the cases, remaining as one of the leading causes of cirrhosis and HCC in Western countries, alongside alcohol consumption [1,2]. In fact, hepatitis C is the main etiology of liver cirrhosis in the United States [1,3,4]. Worldwide, it is estimated that about 71.1 million people have a chronic HCV infection, with a global incidence of 23–7 cases per 100,000 of the population [3,4,5].

Hepatitis C is the result of hepatitis C virus (HCV) infection and, unlike the hepatitis B virus (HBV), causes chronic disease in approximately 50–80% of cases [6]. HCV is mainly transmitted through blood-to-blood contact. It was officially identified in 1989, but, during the 70s, about 90% of post-transfusion hepatitis was attributed to HCV, when it was referred to as non-A, non-B hepatitis. Treatment with interferon-α was initiated shortly thereafter, and, in the 1990s, ribavirin (RBV) was introduced. In 2001, the combination of RBV and pegylated interferon-α (Peg-IFN) became the standard treatment, a protocol that was maintained for almost two decades [7]. In 2011, the first Direct-Acting Antivirals (DAAs) were approved, forever changing the course of HCV chronic infection. Currently, hepatitis C is curable, and the use of DAAs has also demonstrated improvement in extrahepatic manifestations that did not achieve remission or even experience relapse after achieving a sustained virologic response (SVR) [8]. This review provides an overview of the mechanisms underlying these extrahepatic manifestations and the influence of DAAs on them.

## 2. Hepatitis C Virus Biology and Antiviral Targets

As of now, there are four classes of DAAs: NS3/4A protease inhibitors, NS5A replication complex inhibitors, NS5B nucleotide analogue polymerase inhibitors and NS5B non-nucleotide inhibitors (Table 1) [9]. To understand its mechanisms and to understand how HCV spreads its effects beyond the liver, we will briefly explore the HCV life cycle.

HCV is an RNA virus, part of the flaviviridae family, genus Hepacivirus. HCV particles, ranging from 50 to 80 nm in diameter, encapsulate a single-stranded RNA genome, along with core and envelope glycoproteins E1 and E2 [6,7,10]. These glycoproteins are grouped in heterodimers, forming viral spikes on the virion membrane with a size of about 6 nm. A hallmark of HCV morphology is its association with lipoproteins and apolipoproteins like Apo A1, B, C and E, known as lipoviral particles. Regarding its viral determinants, envelope glycoproteins play a vital role in HCV binding and its entry process into the host cell. As with many other viruses, HCV utilizes proteoglycans for attachment; both E1 and E2 form non-covalent heterodimers within the host cells and stabilize the viral particle by disulfide bonds [9,10]. But it is E2 that seems to play a major role, by interacting with CD81 and scavenger receptor B1 (SR-B1). SR-B1 aids viral attachment by interacting with lipoviral particles such as Apo E and the LDL receptor at the early stage of HCV entry. However, it is the CD81 interaction that activates signaling pathways that may be important for the viral infectious cycle. In addition, it is suggested that CD81, along with occludin (OCLN), may explain HCV’s tropism for hepatocytes. In addition, CD81 forms a complex with tight junction protein claudin-1 (CLDN1), involved in the internalization of HCV particles into the host cell in the early steps of HCV entry, while OCLN may help HCV entry in later processes after the initial attachment [10,11,12,13].

In summary, virion entry is followed by the uncoating and dissociation of the viral core, which results in the release of RNA to begin translation and replication within the endoplasmic reticulum (ER). The HCV genome has a length of 9.6 kb and contains a single open reading frame surrounded by two non-translated regions (NTRs). Translation is initiated by one of the NTRs, 5′NTR, by an internal ribosomal entry site (IRES). After translation into a single polyprotein, it is processed by viral and host proteases in ten mature proteins—the core, the structural proteins E1 and E2 and seven non-structural proteins: p7 viroporin, NS2, NS3, NS4A, NS4B, NS5A and NS5B. p7 viroporin and NS2 participate in virus assembly and release; NS3 and NS4A form a protease complex that helps to cleave NS4B, a membrane-associated protein that mediates virus–host interactions; NS5A is a zinc-binding and proline-rich hydrophilic phosphoprotein, key in the RNA synthesis. New virions are assembled in an ER-derived compartment and released by exocytosis, following a Golgi-dependent secretory pathway. Along this process, the virus undergoes maturation and becomes surrounded by endogenous lipoproteins that, as described below, are believed to help immune escape [11,12,13,14].

## 3. Mechanism of Chronic Hepatitis C Virus Infection and Extrahepatic Disease

**Figure 1:**
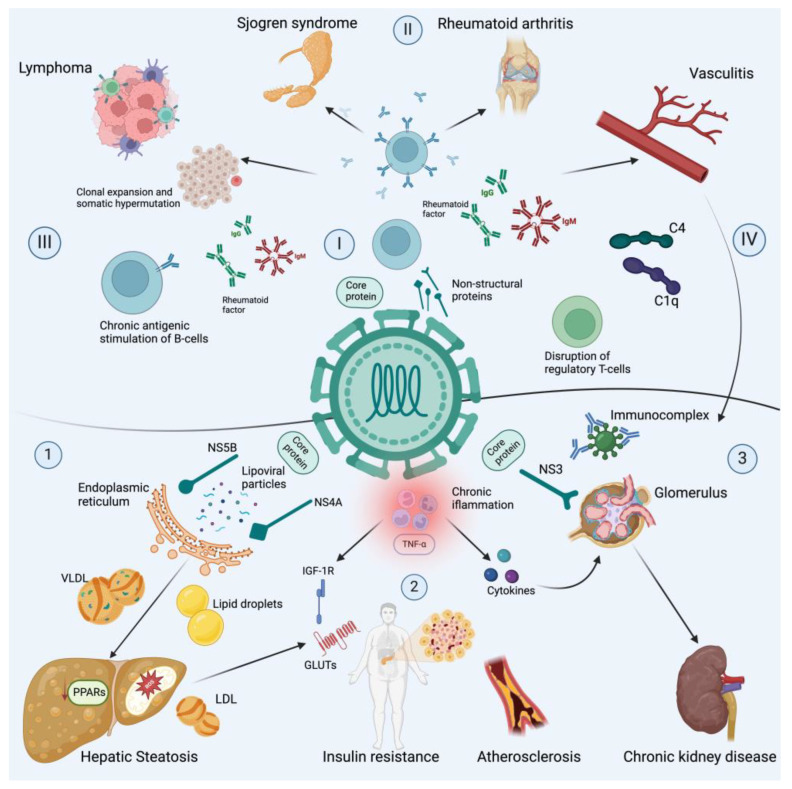
Chronic hepatitis C virus infection and extrahepatic diseases. This figure illustrates the complex relationship between chronic hepatitis C virus (HCV) infection and the development of various extrahepatic diseases. It highlights the main non-hepatic manifestations associated with chronic HCV infection, including immune-related conditions, lymphoproliferative disorders and metabolic alterations. (1) HCV infection disrupts lipoprotein homeostasis, including low-density lipoprotein cholesterol (VLDL). Viral proteins (NS4B and NS5A) induce the formation of lipid droplets (LDs), by altering the endoplasmic reticulum (ER) structure through cellular lipid lipase activation, and ipoviral particles are incorporated into LDs in the VLDL within the ER. In addition, viral proteins downregulate proliferator-activated receptor gamma (PPARγ), which, alongside PPARα, promotes insulin sensitivity and adipogenesis. (2) The latter processes, hepatic steatosis and chronic inflammation characterized by high titers of TNF-α, induce Ser phosphorylation, reducing the activity of insulin receptor substrate 1 (IRS-1) and therefore the expression of glucose transporters (GLUTs), hence inducing insulin resistance and cardiovascular risk by atherosclerosis. (3) Metabolic alterations lead to the development of chronic kidney disease, which may be exacerbated by the local activity of cytokines and deposits of immunocomplexes. Regarding immune mechanisms, the following should be noted: (I) Core and non-structural proteins form cryoglobulins, since they serve as the main ligand for IgM CD5+ B-cells. (II) Chronic antigenic stimulation by the immunocomplexes that are formed between IgG or IgM isotypes and viral antigens are part of the rheumatoid factor origin in the development of SSJ, RA and vasculitis. (III) Furthermore, chronic antigen simulation leads to B-cell clonal expansion; the B-cells in both HCV-MC and lymphomas use the VH1-69 gene and the VkA27 segment, which are at the same time used by anti-E2 antibodies elicited by HCV, supporting the theory of an antigenic selection-driven process underlying lymphoma development. (IV) Immunocomplexes serve as activators for the classical complement pathway by the union of the C1q to CH2 domain of IgG and the CH3 and CH4 of IgM. Even more, the C1q and HCV core bind to gC1qR, with gC1qR/HCV core complexes contributing to C4 deposits in small vessels; this viral interaction may explain renal injury, since the HCV core protein is present within the glomerular and tubulo-interstitial capillaries.

As survival rates have increased in patients with chronic HCV infection, a growing prevalence of extrahepatic manifestations has been observed, many of them asymptomatic. Nevertheless, two-thirds of those patients develop symptoms of HCV-related extrahepatic disease. These conditions can be immune-related conditions, lymphoproliferative disorders and even inflammatory diseases that may disrupt many metabolism routes, like lipids and insulin resistance (IR) (Figure 1) [15,16]. Less common conditions include Sjögren’s syndrome (SSJ) and fibromyalgia [16,17].

### 3.1. Autoimmune Manifestations

Autoimmunity emerged as the initial extrahepatic manifestation linked to HCV, with mixed cryoglobulinemic vasculitis (HCV-CV) being the most prevalent immune-related extrahepatic condition in chronic HCV infection. This condition, specifically Types II and III, is marked by vasculitis affecting small vessels, triggered by polyclonal IgM or IgG and characterized by a positive rheumatoid factor (RF). While in most cases HCV-CV is asymptomatic, up to 10% of patients develop symptoms like peripheral arthralgias, palpable purpura and neuropathy, the latter being a common and persistent symptom even after SVR [14,18,19]. A lesser proportion of those patients will develop renal involvement, which is perhaps the most severe presentation; this membranoproliferative glomerulonephritis has a 10-year survival of 63% [16].

The main mechanism of HCV-CV involves the interaction between HCV, B-cells and regulatory T-cells, where continuous stimuli for the immune cells in the setting of chronic infection affects their capacity to produce neutralizing antibodies, resulting in polyclonal activation and the expansion of B-cells producing IgM with RF activity, a critical event for the cryoprecipitating process [19,20]. In fact, the HCV core protein seems to be the most important element in forming cryoglobulins, since it serves as the main ligand for IgM CD5+ B-cells [18]. In addition, autoimmune modulation seems to be disrupted, since regulatory T-Cells (CD4 + and C25+) are decreased [20].

The mechanism behind the formation of RF is still not completely understood, but it may be attributed to chronic antigenic stimulation by the immunocomplexes that are formed between IgG or IgM isotypes and viral antigens. In addition, those same immunocomplexes serve as activators for the classical complement pathway, by the union of C1q to the CH2 domain of IgG and to the CH3 and CH4 of IgM. Even more, C1q and the HCV core bind to gC1qR, with gC1qR/HCV core complexes contributing to C4 deposits in small vessels; this viral interaction may explain renal injury, since the HCV core protein is present within the glomerular and tubulo-interstitial capillaries [18,19,21,22]. Immunocomplexes, RF and their complements serve as important cyroprecipitable components.

Following HCV autoimmune and lymphoproliferative activity, it is well documented that the overall risk of developing B-cell lymphoma is higher when compared with the general population, and even more so when HCV-CV is present; approximately 8–10% of those patients develop lymphoproliferative disorders [15,20,23]. The predominant types are non-Hodgkin lymphoma (NHL), particularly low-grade marginal zone lymphoma (MZL), representing about 12% of B-cell lymphomas, followed by diffuse large B-cell lymphoma (DLBCL) [23,24]. HCV-MZL mainly originates from the spleen and occurs after a course of more than 15 years of HCV chronic infection, with an indolent development independent from liver fibrosis and diagnosed in older patients, about 65 years old [23,24,25]. As with HCV-mixed cryoglobulinemia (HCV-MC), it is proposed that HCV lymphomas develop after chronic antigenic stimulation by HCV, leading to B-cell mutation and/or bcl rearrangement [15,24].

As stated above, HCV-MC is a major risk factor for NHL, with the risk increased by about 35 times [23,25]. Furthermore, chronic antigen simulation leads to B-cell clonal expansion; B-cells in both HCV-MC and lymphomas use the VH1-69 gene and VkA27 segment, which are at the same time used by anti-E2 antibodies elicited by HCV, supporting the theory of an antigenic selection-driven process underlying lymphoma development. In addition, HCV-MC immunoglobulins and RF are associated with other NHLs such as MALT lymphoma, and immunoglobulins may be a result of somatic hypermutation [20,24,25,26]. In the case of MZL, HCV’s antigenic stimulation of B-cells in the spleen by the E2 viral protein binds to the CD81 receptor of the B-cell, inducing somatic hypermutation [20,25,26]. Therefore, the elimination of antigenic stimuli may reduce the risk of lymphoproliferation. Even before the use of DAAs, reaching an SVR with RBV and Peg-IFN in patients with HCV-MC showed no occurrence of B-cell lymphoma in a post-treatment mean follow-up of 92.5 months [26].

Regarding less common associations, only less than 5% of patients develop SSJ, although some series and cohort studies report a prevalence of about 13%. Interestingly, an important proportion of those patients develop sicca symptoms, despite a lower frequency of anti-Ro/La antibodies [15,27,28]. This same phenomenon occurs in arthritis, where primary rheumatoid arthritis is rare and most of arthritis cases have a negative anti–cyclic citrullinated peptide but positive RF, and it develops in the context of HCV-CV [29].

### 3.2. Metabolic Manifestations

Regarding other effects, the current evidence has shown significant metabolic alterations in chronic HCV infection. For instance, a prevalence is estimated of about 55.54% for liver steatosis in patients with HCV infection, especially when infected with genotype 3, increasing fibrosis progression with pre-existing liver steatosis but also as a bad response to antiviral therapy [30,31]. HCV infection disrupts lipoprotein homeostasis, including the low-density lipoprotein cholesterol (VLDL)-releasing pathway. In fact, viral proteins like NS4B and NS5A induce the formation of lipid droplets (LDs) by altering the ER structure through cellular lipid lipase activation [30,32]. Furthermore, lipoviral particles are incorporated into LDs in the VLDL within the ER [31,32]. Other mechanisms have been proposed, but liver steatosis is not the only reason why HCV increases cardiovascular risk.

HCV pro-inflammatory effects extend also to glucose metabolism, leading to IR. In fact, patients with chronic HCV infection have an 11.5 times higher risk of developing Type 2 diabetes mellitus (T2DM) [33]. Moreover, Desbois and colleagues concluded in a meta-analysis that patients infected with HCV presented more glucose abnormalities, a lack of SVR and a higher risk of HCC development when compared with patients infected with HBV [34]. While the HCV genotype 3 viral load seems to increase liver steatosis prevalence, genotypes 1,2 and 4 are more related to liver steatosis due to a metabolic syndrome, rather than direct impairing the lipid metabolism in the liver [31,34]. For instance, HCV genotype 1 core proteins downregulate proliferator-activated receptor gamma (PPARγ), which, alongside PPARα, promotes insulin sensitivity and adipogenesis [34,35]. Yet, viral steatosis by HCV genotype 3, or metabolic alterations due to non-3 genotypes, perpetuates liver fibrosis through IR and inflammatory cytokines [31]. Moreover, high titters of TNF-α are present in HCV chronic infection, and this cytokine induces Ser phosphorylation, reducing the activity of insulin receptor substrate 1 (IRS-1) and therefore the expression of glucose transporters (GLUTs) [34,36,37,38].

Either via dyslipidemia or IR, cardiovascular risk is increased in HCV chronic infection. Early studies showed a significant prevalence of atherosclerosis in this population [36]. A meta-analysis in 2016 reported that, in contrast to controls, patients with HCV had an elevated risk of cardiovascular mortality, the presence of carotid plaques (OR, 2.27; 95% CI, 1.76–2.94 *p* < 0.001) and cerebrocardiovascular events (OR, 1.30; 95% CI, 1.10–1.55; *p* = 0.002) [39]. Nevertheless, a reduction in cardiovascular outcomes is possible by using antiretroviral therapy, according to a recent meta-analysis that examined alterations in IR, measured by HOMA-IR levels, subsequent to successful HCV eradication through DAA therapy across 23 studies. On the other hand, while this study underscores the benefits of an SVR on IR, certain limitations exist, including the lack of individual patient data on demographics and genotype distribution across the studied populations. Additionally, a reliance solely on HOMA-IR may overlook other markers of IR that warrant further investigation [40].

### 3.3. Neuropsychiatric Manifestations

Beyond the significant impact on cardiovascular health, HCV infection is also associated with a wide range of neuropsychiatric manifestations, reflecting the complex interaction between the virus, the immune system and the central nervous system (CNS) [41]. Cognitive dysfunction is a common neuropsychiatric manifestation among individuals with chronic HCV infection. Patients may experience difficulties with concentration, attention, memory and executive functions. These cognitive disturbances, often referred to as “brain fog” [42,43]. Furthermore, depression, anxiety and fatigue are also common among HCV patients. Studies have shown that individuals with HCV infection have an increased risk of developing mood disorders compared to the general population [44,45]. The molecular mechanisms underlying the neuropsychiatric manifestations of HCV infection involve a complex interplay of viral factors, immune responses and their effects on the CNS [44]. HCV can permeate the blood–brain barrier (BBB), resulting in the direct infection of CNS cells, such as astrocytes and microglia. This direct invasion of the virus can cause neuro-inflammation and neurodegeneration [42]. Molecular studies have identified HCV RNA in the brain, suggesting that the virus can replicate in the CNS and trigger a cascade of neuro-inflammatory responses [46]. Additionally, the immune response to HCV infection plays a crucial role in the neuropsychiatric manifestations of the disease. Chronic HCV infection causes systemic inflammation, characterized by the release of proinflammatory cytokines such as IL-6 and TNF-α [47]. These cytokines can penetrate the BBB and affect the CNS, leading to changes in neurotransmitter systems, neuro-inflammation and alterations in neurocircuitry that are related to mood and cognitive functions [48].

### 3.4. Kidney Manifestations

Type I membranoproliferative glomerulonephritis (MPGN) is the most common kidney consequence among patients with HCV-associated cryoglobulinemia (HCV-CV), affecting up to 55% of patients [15]. This condition is characterized by significant alterations in the filtration system of the kidney, specifically in the glomeruli, leading to varying degrees of kidney dysfunction [49]. While MPGN with cryoglobulinemia represents a direct immune response to the virus, HCV can also instigate renal diseases such as MPGN without cryoglobulinemia and membranous glomerulonephritis, although less frequently. These conditions, while varying in their pathophysiologic origins and clinical presentations, highlight the diverse kidney manifestations associated with HCV infection [50]. The mechanisms by which HCV induces kidney disease are multifaceted. Immune complexes, formed in response to the virus, can be deposited in the glomeruli, triggering inflammation and subsequent tissue damage [51]. In addition, the ability of HCV to directly invade kidney tissue adds another layer of potential damage, contributing to the spectrum of renal pathology seen in infected individuals. Complications in the kidneys may also arise indirectly, as a result of the impact of HCV on other organs, especially the liver, reflecting the interconnectedness of organ function within the body [52]. In addition, the drug treatment of HCV can have nephrotoxic effects, which poses challenges in the management of infected patients and requires careful consideration of therapeutic strategies to minimize renal damage [53].

### 3.5. Cancer Manifestations

In addition, HCV infection increases the risk of extrahepatic cancers besides NHL. Different population studies report an incidence of breast, thyroid cancer, pancreatic cancer, gallbladder cancer, kidney cancer, lung cancer and oropharyngeal cancer. Nevertheless, none of them has a significant association, and the underlaying mechanisms are unclear [54,55,56]. In this matter, a large French study investigated the impact of DAAs on cardiovascular events and extrahepatic cancers. It was observed that DAAs were not associated with extrahepatic cancer development or reduction. On the other hand, the reduction in cardiovascular risk seemed to be related with the reduction of other factors such as metabolic pathways or improving fibrosis, but there was an increase in cardiac arrhythmias, especially in SOF-based regimes [57].

### 3.6. Deficiency of Vitamin D

Finally, there are other associations and mechanism of HCV extrahepatic manifestations. Several studies correlate a deficiency of vitamin D in the setting of HCV chronic infection with the development of vasculitis and NHL [58,59,60]. It has been proposed that vitamin D plays a pivotal role in immune system modulation by facilitating the generation of regulatory T-cells and reducing the levels of pro-inflammatory cytokines like Th1 and Th17 cytokines, as well as inhibiting B-cell differentiation. The potential benefits of vitamin D supplementation in managing inflammatory responses and fibrogenesis have been suggested in chronic HCV infection. Recent research on genotype 1 HCV-mono-infected individuals has indicated associations between low serum 25(OH)D levels and severe liver fibrosis and a diminished SVR, supporting the idea that the supplementation with vitamin D of antivirals improves treatment efficacy [58,59].

More impacts of DAAs on metabolic alterations and other extrahepatic manifestations are described further.

## 4. Impact of Direct-Acting Antiviral Agents on Extrahepatic Disease

Perhaps the first study to explore the effects of DAAs on metabolic alterations was that published by Hashimoto et al. in 2016, using two different combinations of the NS3/4A protease inhibitor, plus a NS5A replication complex inhibitor. A total of 24 patients underwent DAC/ASV combination therapy for 24 weeks, and 76 patients who underwent LDV/SOF for 12 weeks showed an increase in LDL concentration and in total cholesterol, with no effect on HDL cholesterol levels [61]. In addition, when comparing DAAs with older therapies like Peg-IFN/RBV after reaching an SVR, it seems that DAAs increase LDL levels with no effects on IR [62]. Similar findings were reported in a more recent study, with different combinations between LDV, SOF, OBV, PTV, RBV and ritonavir. During treatment, there was an observed rise in insulin resistance (IR), total cholesterol (TC) and low-density lipoprotein (LDL) cholesterol levels. Following the cessation of treatment, IR returned to baseline levels, while the heightened TC and LDL levels remained elevated indefinitely [63]. In contrast, at least two studies reported an improvement in endothelial function and a reduction in atherosclerosis after DAA regimens, reducing cardiovascular risk [64,65]. While the positive impact of DAAs on lipid metabolism cardiovascular risk remains, there is no doubt that the new antiretrovirals reduce cardiovascular mortality by the achievement of an SVR, since it is linked to enhanced cardiovascular outcomes, eliminating various other adverse effects of HCV infection. For instance, more recent studies showed that, despite IR persisting, as SVR following DAAs lowers fast glucose, insulin levels and IR, even in patients with HCV liver cirrhosis [66,67]. Moreover, DAAs improve renal function in patients with chronic kidney disease (CKD), T2D and HCV chronic infection; while serum creatinine did not change significantly at any time, the eGFR significantly improved after 12 weeks of DAA therapy [68]. In general, reaching an SVR improves renal function [69].

Regarding lymphoproliferative disorders, the clearance of HCV reduces the rates of HCV-CV and B-cell lymphoma [70]. Before DAAs, HCV-CV patients experienced relapse under treatment with Peg-IFN/RBV, even after reaching an SVR [71]. Several studies have explored the effects of DAAs in HCV-CV, with and without a specific CV like Rituximab. The largest study included 148 patients, in which 95% of the patients had a full or partial response of symptoms to different DAA treatment regimens. It included four different combinations: SOF/DAC, SOF/RBV, SOF/LDV and SOF/SIM [72]. In another successful study, 41 patients who underwent DAA therapy with SOF/DAC achieved, as in the previous one, important remissions of purpura, neuropathy and arthralgia [73]. Some other studies showed similar outcomes [74,75]. On the other hand, an SVR with DAAs may improve HCV-CV symptoms at six months, but patients could relapse at one year after antiretroviral therapy [76,77].

A recent study demonstrated that treatment with DAAs not only targets the viral load effectively but also has a profound impact on the neuropsychiatric manifestations associated with HCV infection. The findings suggest that DAA therapy is associated with a lower risk of developing new cases of neuropsychological disorders compared to interferon-based regimens, particularly among treatment-naïve HCV patients [78].

In a comprehensive systematic review, a remarkable relationship was found between achieving an SVR and a reduced occurrence of kidney disease. Analyses aggregating viral response outcomes indicated that patients who achieved as SVR experienced a significantly lower risk of developing kidney disease, with the overall adjusted risk reduction measured as 2.50 (95% CI, 1.41–4.41; *p* = 0.0016). In addition, a comparison between treatment and untreated cohorts revealed that antiviral therapy contributed to a reduced risk of kidney disease, with a summary adjusted risk across all studies of 0.39 (95% CI, 0.25–0.612; *p* = 0.0001) [69].

Meta-regression analysis further highlighted that the effectiveness of antiviral therapy in reducing the frequency of kidney disease decreases as cirrhosis and HBV infection increase among HCV-infected individuals.

In the case of B-cell lymphoma, DAA regimes, particularly SOF-based regimes, prove to be effective in obtaining the hematological response of lymphoma [79]. One of the first studies was a case series of patients with chronic HCV infection with MZL or DLBCL, where all achieved a hematological response and SVR after a one-year follow-up [80]. Two more recent studies with larger samples reported similar but less empathic results. In one of them, only 21% of patients with MZL achieved a complete hematological response, while in the other study, 98% of patients with DLCBL achieved a complete hematological response and 96% an SVR [81,82]. In contrast, a more recent study concluded that DAAs may serve as a first-line therapy for indolent lymphomas, but more studies are needed to support that statement [83]. Finally, DAAs are shown to be safe and effective in the remission of B-cell lymphomas in HCV patients [84].

## 5. Conclusions

With the advent of DAAs, hepatitis C is now curable. Furthermore, their impact goes beyond that by improving extrahepatic alterations. The prevalence of symptomatic non-hepatic disease may be low when compared with the general prevalence of such manifestations, but when symptoms develop, their outcomes are disastrous, beginning with the development of indolent NHL and its bad prognosis, even with concomitant chemotherapy. In general, it seems that DAAs have a positive impact in reducing the incidence of MZL and achieving a partial or complete hematological response alongside an SVR. Following these lymphoproliferative disorders, the treatment of DAAs in the setting of HCV-CV is better than the old antivirals and, even without comparing, improves symptoms and reduces morbidity. On the other hand, there are contrasting results among studies about the relapse of HCV-CV, since patients improve at six months but relapse after one year of treatment, even with an SVR. In this matter, few studies approach the possibility of fluctuating symptoms, and most of them have a one-year follow-up. Yet, most of the persistent symptoms include arthralgia and neuropathy, while DAAs may cure major affections like renal injury. More controversial is the impact of DAAs on the metabolic syndrome, particularly in the lipid metabolism, because of the elevation of LDL but the reduction of triglycerides. This could result, paradoxically, in the rising in LDL levels responding to adaptive changes after HCV clearance, while the reduction in triglycerides is the consequence of the elimination of direct inflammatory stimuli in the liver. In addition, the improvement in triglycerides affects HE, therefore reducing cardiovascular risk. In the case of IR and T2D, it is important to highlight the fact that, despite IR persisting, it actually improves, as well as fasting glucose. Moreover, the reduction in eGFR due to an SVR is enhanced by the improving IR; this could explain that DAAs reduce cardiovascular risk by improving other metabolic alterations and not only through lipid metabolism. Finally, in general, the role of DAAs is not only to cure hepatitis C but also to serve as an adjuvant therapy of extrahepatic disease; some patients will need concomitant therapies like chemotherapy or rituximab, not to mention the baseline treatment for T2D or dyslipidemia.

## Figures and Tables

**Table 1:** Approved Direct-Acting Antivirals per group for hepatitis C virus treatment.

NS3/4A Protease Inhibitor	NS5B Nucleotide Analogue Inhibitor	NS5B Non-nucleotide Inhibitor	NS5A Replication Complex Inhibitors
Simeprevir (SIM)	Sofosbuvir (SOF)	Dasabuvir (DSV)	Daclatasvir (DAC)
Asunaprevir (ASV)			Ombitasvir (OBV)
Paritaprevir (PTV)			Ledipasvir (LDV)
Grazoprevir (GZR)			Elbasvir (EBR)
Voxilaprevir (VOX)			Velpatasvir (VEL)
Glecaprevir (GLE)			Pibrentasvir (PIB)

Main combinations include: LDV/SOF, EBR/GZR, SOF/VEL, SOF/VEL/VOX, GLE/PIB.
